# Systematic review of behavior-related self-management assessment instruments for patients with diabetes

**DOI:** 10.3389/fpubh.2026.1858702

**Published:** 2026-08-28

**Authors:** Jun Zhou, Yuanyuan Cui, Xingxin Cai, Mengqi Han, Wenxiu Wang, Peimin Liu, Jing Li, Guiying Guo

**Affiliations:** 1 School of Nursing, Shanxi University of Chinese Medicine, Jinzhong, Shanxi, China; 2 The Second Ward of Endocrinology, Third Clinical College, Shanxi University of Chinese Medicine, Taiyuan, Shanxi, China

**Keywords:** assessment instruments, diabetes mellitus, measurement properties, self-management, systematic review

## Abstract

**Objective:**

To systematically evaluate the methodological quality and measurement properties of behavior-related self-management assessment instruments for patients with diabetes, and to provide evidence-based guidance for healthcare professionals when selecting appropriate assessment instruments.

**Methods:**

A systematic literature search was conducted in CNKI, Wanfang Database, VIP Database, China Biomedical Literature Database (CBM), PubMed, Web of Science, Embase, and CINAHL to retrieve studies on behavior-related self-management assessment instruments for patients with diabetes. The search timeframe spanned from the inception of each database to December 15, 2025. Two independent researchers conducted literature screening and data extraction in accordance with predefined inclusion and exclusion criteria. The COSMIN guideline was used to evaluate the methodological quality of the included studies and the measurement properties of the included instruments and to formulate recommendation levels.

**Results:**

A total of 14 studies involving 11 assessment instruments were included. No instrument met the criteria for a Grade A recommendation; 5 instruments were classified as Grade B and 6 as Grade C. Overall, the methodological quality and psychometric evidence base of the included instruments were limited. Content validity was frequently rated as indeterminate due to insufficient methodological reporting, while evidence for cross-cultural validity, measurement error, and responsiveness was largely absent. Among the included instruments, the SDSCA showed relatively stronger evidence for adults with type 2 diabetes mellitus, and the C-SMOD-A-23 showed relatively stronger evidence for adolescents with type 1 diabetes mellitus. However, both instruments were rated as Grade B and should therefore be used with caution rather than strongly recommended.

**Conclusion:**

Evidence supporting the psychometric performance of diabetes self-management behavior scales is limited. While SDSCA and C-SMOD-A-23 could be used for select groups, their application requires caution due to unclear content validity and inadequate evidence of critical measurement properties. Future validations should follow COSMIN standards and prioritize content validity, cross-cultural validity, measurement error and responsiveness. Owing to absent age-specific validation data, existing tools’ suitability for older adults with diabetes—especially those with frailty, multimorbidity, cognitive dysfunction, functional/sensory impairment, polypharmacy and caregiver dependence—remains unclear and warrants targeted further research.

**Systematic review registration:**

https://www.crd.york.ac.uk/PROSPERO/view/CRD420251178394, identifier: CRD420251178394.

## 1 Introduction

According to the International Diabetes Federation (IDF), the rising prevalence of diabetes has become one of the major global public health challenges (1). In 2021, approximately 529 million people worldwide were living with diabetes, and this number is projected to rise to 1.31 billion by 2050 (2). As diabetes is a chronic and incurable disease, individuals with diabetes rely on long-term medical treatment and consistent self-management to slow disease progression. In this review, diabetes-related self-management behaviors are defined as the self-care actions undertaken by people with diabetes to regulate blood glucose levels and reduce the risk of complications. These behaviors include dietary regulation, physical activity, blood glucose monitoring, medication or insulin management, foot care, hypoglycemia and hyperglycemia management, and follow-up or re-examination behaviors (3). Instruments that assess only diabetes-related knowledge, attitudes, self-efficacy, health beliefs, or diabetes distress were therefore considered conceptually distinct from tools that assess actual self-management behaviors. Evidence from relevant studies indicates that effective self-management strategies can improve glycemic control, reduce mortality, enhance health-related quality of life, and lower healthcare costs (4–6). Accordingly, accurately assessing behavior-related self-management capacity among people with diabetes is critical for developing personalized, targeted diabetes management interventions. Population ageing further increases the complexity of diabetes management (2, 7). Older adults with diabetes often experience frailty, multimorbidity, cognitive impairment, functional decline, sensory limitations, polypharmacy, and varying degrees of dependence on spouses, adult children, or formal caregivers (8–12). These age-related factors may substantially influence self-management behaviors, including dietary regulation, physical activity, blood glucose monitoring, medication or insulin management, foot care, recognition and management of hypo- or hyperglycemia, and attendance at follow-up visits (8–11). Therefore, instruments developed or validated in general adult populations may not fully capture the feasibility, complexity, and support needs of self-management among older adults with diabetes (8–10).

Currently, a variety of assessment instruments exist for behavior-related diabetes self-management, yet they vary widely in quality and psychometric performance. Many instruments lack validation across diverse populations and theoretical grounding, creating substantial challenges in selecting appropriate measurement tools for clinical practice. The Consensus-Based Standards for the Selection of Health Measurement Instruments (COSMIN) (13) provides a rigorous framework for evaluating such tools, covering content validity, structural validity, internal consistency, criterion validity, hypothesis testing, reliability, measurement error, cross-cultural validity, and responsiveness.

Although previous studies have reviewed diabetes self-management assessment instruments, existing reviews have several limitations. Some focused primarily on the number and types of available scales (14), some targeted only specific language versions (15), and others included instruments measuring constructs such as knowledge, attitudes, self-efficacy, health beliefs, or diabetes distress (16–17). These constructs are conceptually related to, but distinct from, the actual performance of self-management behaviors. Therefore, a focused evaluation of behavior-related diabetes self-management instruments using the COSMIN framework remains necessary.

This systematic review aimed to evaluate the methodological quality and measurement properties of behavior-related diabetes self-management assessment instruments using the COSMIN guideline, and to provide evidence-based guidance for selecting currently available instruments for different populations and clinical or research contexts. Although this review was not restricted to older adults, the findings may help clarify whether current behavior-related diabetes self-management instruments provide adequate evidence to support their use in ageing populations.

## 2 Materials and methods

### 2.1 Study design

The protocol for this systematic review was registered in the International Prospective Register of Systematic Reviews (PROSPERO) under registration number CRD420251178394. No amendments were made to the registered protocol. This systematic review was reported in accordance with the PRISMA-COSMIN for OMIs 2024 guideline (18).

### 2.2 Inclusion and exclusion criteria for literature selection

Inclusion criteria were as follows: ① Study participants were patients with diabetes; ② The research topic focused on the assessment of behavior-related self-management or self-care capacity in this population; ③ The study content involved the evaluation of at least one COSMIN psychometric property; ④ Study types included the development, revision, and cross-cultural adaptation of measurement instruments; ⑤ Multidimensional instruments with both behavioral and non-behavioral components were included only when actual self-management behavior represented the primary construct and most items reflected behavioral performance.

Exclusion Criteria were as follows: ① Scales measuring only a single domain of self-management (e.g., physical activity, medication adherence, dietary management); ② Scales used solely as outcome assessment instruments or for criterion validity testing; ③ Instruments assessing only non-behavioral constructs, such as knowledge, attitudes, self-efficacy, health beliefs, or diabetes distress; ④ Non-Chinese and non-English publications; ⑤ Duplicate publications or studies with unobtainable full texts; ⑥ Conference abstracts, reviews, and commentaries.

### 2.3 Literature search strategy

A systematic electronic search was conducted to identify studies on assessment instruments for behavior-related self-management in patients with diabetes. The databases searched included China National Knowledge Infrastructure (CNKI), Wanfang Database, VIP Database, Chinese Biomedical Literature Database (CBM), PubMed, Web of Science, Embase, and CINAHL. The search period covered from the inception of each database to December 15, 2025. Full search strategies for all databases are provided in Supplementary figures. In addition, the reference lists of all included studies and relevant reviews were manually searched to identify potentially eligible studies. Searches were restricted to Chinese and English publications because these were the languages in which the review team could reliably assess full texts and COSMIN methodological details. All searches were performed using a combination of controlled subject headings and free-text terms. In addition, the reference lists of all included studies were manually searched to identify potentially eligible publications. Taking CNKI as an example, the Chinese search string is as follows: (Subject: diabetes mellitus OR type 1 diabetes OR type 2 diabetes) AND (Title/Keyword/Abstract: self-management OR self-care) AND (questionnaire OR scale OR instrument) AND (reliability OR validity OR psychometric property). The English search terms are listed as follows: Population: Diabetes Mellitus, Diabetes Mellitus Type 2, Type 2 Diabetes, Diabetes Type 2, Type 2 Diabetes Mellitus, Diabetes Mellitus Type II, Diabetes Mellitus Stable, Adult-Onset Diabetes Mellitus, Maturity-Onset Diabetes, Diabetes Mellitus Type 1, Type 1 Diabetes, Diabetes Type 1, Diabetes Mellitus Type I, Ketosis-Prone Diabetes Mellitus. Construct: Self-Management, Self Management, Management Self, Self-Management Programs, Self-Management Program, Self Care. Instrument: questionnaire*, scale, instrument*, tool*. Psychometric properties: reliab*, valid*, psychometr*. The search strategy is based on PubMed as an example (Supplementary Figure 1).

### 2.4 Literature screening and data extraction

Two reviewers independently conducted literature screening, data extraction, and methodological quality assessment. Prior to formal evaluation, both reviewers received training on the COSMIN Risk of Bias Checklist. They conducted a pilot test of the extraction and scoring procedures using a small sample of studies to enhance rating consistency. A standardized data extraction form developed in accordance with the COSMIN guidelines was used throughout the review. Any disagreements regarding study inclusion, data extraction, or COSMIN ratings were first resolved via discussion. If consensus could not be reached, a third senior reviewer was consulted. This procedure was implemented to ensure methodological transparency and consistent application of the COSMIN criteria.

Literature management was performed using EndNote. Two reviewers independently screened records by titles and abstracts, followed by full-text assessment for potentially eligible studies. Extracted data included the name of the assessment instrument, first author, publication year, country/region, sample size, number of domains and items, target population, and other key characteristics.

### 2.5 Quality assessment

#### 2.5.1 Methodological quality assessment

The methodological quality of the included studies was assessed using the COSMIN Risk of Bias checklist (19). The evaluated domains included instrument development, content validity, structural validity, internal consistency, reliability, measurement error, criterion validity, hypothesis testing for construct validity, cross-cultural validity, and responsiveness. Because all included instruments were self-report questionnaires, the reliability evidence focused mainly on test–retest reliability. Each item within each domain was rated on a four-point scale: “very good,” “adequate,” “doubtful,” or “inadequate”. In accordance with the “worst score counts” principle, the lowest item score within each domain determined the overall methodological quality rating for that domain.

#### 2.5.2 Quality assessment of measurement properties

The measurement properties of the included instruments were evaluated according to the COSMIN criteria for good measurement properties (20). Content validity was assessed first, followed by the remaining measurement properties. If high-quality evidence indicated insufficient content validity, assessment of the remaining properties was discontinued. The result for each measurement property was classified as “sufficient”, “insufficient”, or “indeterminate”. If results for a given property were consistent across studies, an overall rating was summarized and reported. If results were inconsistent and could not be explained, the overall rating for that property was classified as “inconsistent”.

#### 2.5.3 Evidence level assessment and recommendations

The quality of evidence was evaluated using the modified grading system for quantitative systematic reviews, which classifies evidence quality as high, moderate, low, or very low (21). An initial high-quality rating was assigned, and the evidence quality for each measurement property was downgraded according to risk of bias, inconsistency, imprecision, and indirectness. Recommendation levels were determined according to COSMIN guidance. A Grade A recommendation was assigned if an instrument showed sufficient content validity and sufficient internal consistency supported by at least low-quality evidence. A Grade C recommendation was assigned if high-quality evidence indicated insufficient performance for at least one measurement property. All other eligible instruments were classified as Grade B.

## 3 Results

### 3.1 Literature search results

**Figure 1:**
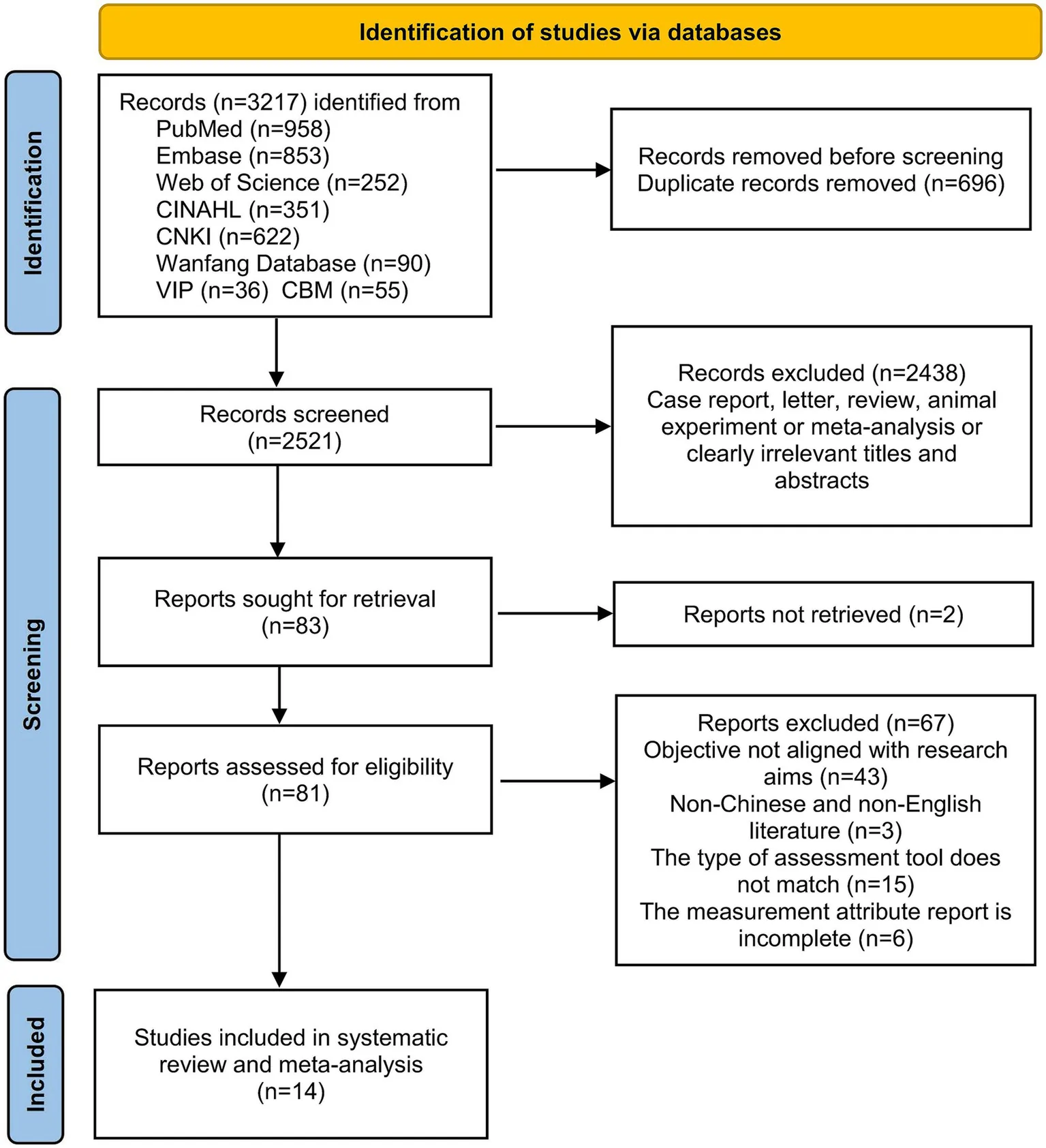
Study search and selection flowchart.

A total of 3,217 records were initially identified through database searching. After removing 696 duplicate records, 2,438 records were excluded following title and abstract screening against predefined inclusion and exclusion criteria. Following full-text assessment, a further 83 records were excluded. Finally, 14 studies (22–35) were included in this systematic review. The flowchart of study selection is presented in Figure 1.

### 3.2 Basic characteristics of the included assessment instruments

Among the 14 included studies (22–35), 11 distinct assessment instruments for behavior-related self-management in patients with diabetes were identified. Specifically, 6 studies (22, 24, 26, 29, 33, 35) reported cultural adaptation and validation of existing instruments; 4 studies (25, 27, 28, 30) described the development of new self-management assessment instruments; 2 studies (23, 34) involved the revision of established instruments; and 2 studies (31, 32) validated short-form versions of existing scales in local populations. The basic characteristics of the included studies are summarized in Table 1. Regarding age-specific applicability, none of the included studies specifically targeted older adults with diabetes as the primary validation population.

**Table 1:** Basic characteristics of included studies.

Included studies	Publication year	Instrument name	Country	Retest interval	Target population	Sample size	Number of domains/items	Response options
Oliveira D (22)	2024	DSMQ-R	Portugal	2 weeks	T2DM	365	5/27	Likert 4
Schmitt A (23)	2021	DSMQ-R	Germany	3–6 months	T1DM and T2DM	1,447	5/27	Likert 4
Li CQ (24)	2018	DSMQ	China	NR	DM	370	4/16	Likert 4
Ausili D (25)	2017	SCODI	Italy	NR	T2DM	200	4/40	Likert 5
Wang X (26)	2021	C-SCPI	China	2 weeks	T2DM	375	3/23	Likert 7
Mbuagbaw L (27)	2017	SCPI	Canada	6 months	T1DM and T2DM	120	3/25	continuous scoring from 0 to 10
Dai Z (28)	2023	DSMS	China	NR	T2DM	469	3/22	Mixed response formats: dichotomous, 3-point, and 5-point Likert options
Şahin Yildiz Y (29)	2023	CDSMS	Turkey	NR	T2DM	475	5/13	mixed scoring of 0–4 points (multiple choice) and 1 point (dichotomous items)
Zhu S (30)	2020	SMOD-CA	China	2 weeks	T1DM	243	4/30	Likert 5
Lee S L (31)	2018	C-SMOD-A-23	China	2 weeks	T1DM	200	4/23	Likert 4
Lee C L (32)	2016	DSMI-20	China	2 weeks	T2DM	232	4/20	Likert 4
Hua L (33)	2014	SDSCA	China	4 months	T2DM	419	5/11	behavioral frequency in the past 7 days, some items scored separately
Khagram L (34)	2013	SCI-R	UK	NR	T2DM	353	1/13	Likert 5
Tang XR (35)	2025	SCODI	China	NR	DM	261	4/40	Likert 5

This table summarizes the main characteristics of the included studies, including publication year, instrument name, country, retest interval, target population, sample size, number of domains/items, and response format. NR = not reported; DM = diabetes mellitus; T1DM = type 1 diabetes mellitus; T2DM = type 2 diabetes mellitus.

### 3.3 Results of methodological quality and measurement properties assessment for the included instruments

None of the 14 included studies (22–35) reported data on the cross-cultural validity of the assessment instruments. The results of methodological quality assessment are presented in Table 2, and the results of measurement properties assessment are summarized in Table 3.

**Table 2:** Methodological quality ratings and study-level measurement property results of the included studies.

Included studies	Scale development and evaluation	Content validity			Structural validity	Internal consistency	Test–retest reliability	Criterion validity
		Relevance	Comprehensibility	Comprehensiveness				
Oliveira D (22)	D	D	D	NR	V/+	V/−	A/+	NR
Schmitt A (23)	NR	NR	NR	NR	V/+	V/−	I/?	V/−
Li CQ (24)	D	D	D	D	V/−	V/−	NR	NR
Ausili D (25)	D	D	NR	D	A/+	V/+	NR	NR
Wang X (26)	D	D	D	D	A/−	V/+	D/?	V/−
Mbuagbaw L (27)	D	D	NR	D	I/?	V/+	I/?	NR
Dai Z (28)	D	D	NR	NR	V/−	V/+	NR	I/−
Şahin Yildiz Y (29)	D	D	D	NR	V/+	V/−	NR	V/−
Zhu S (30)	D	D	NR	D	A/?	V/+	D/?	I/−
Lee S L (31)	D	D	NR	D	V/+	V/+	A/+	I/−
Lee C L (32)	D	D	D	D	A/−	V/+	D/?	NR
Hua L (33)	D	D	NR	NR	V/+	V/+	I/?	I/−
Khagram L (34)	NR	NR	NR	NR	V/−	V/+	NR	NR
Tang XR (35)	D	D	D	NR	A/?	V/+	NR	I/−

This table presents study-level COSMIN methodological quality ratings and measurement property results for each included study. For structural validity, internal consistency, test–retest reliability, and criterion validity, the first symbol indicates methodological quality and the second symbol indicates the rating of the measurement property result. Methodological quality ratings were based on the COSMIN Risk of Bias checklist: V = very good; A = adequate; D = doubtful; I = inadequate; NR = not reported. Measurement property results were rated as + = sufficient; − = insufficient; ? = indeterminate. Relevance, comprehensibility, and comprehensiveness refer to the three components of content validity. NR = not reported.

**Table 3:** Summary of measurement property results, evidence quality, and COSMIN recommendation levels for the included assessment instruments.

Instrument name	Content validity	Structural validity	Internal consistency	Test–retest reliability	Criterion validity
DSMQ-R (16, 23)	?/Moderate	+/High	−/High	?/Very low	−/High
DSMQ (24)	?/Low	−/High	−/High	NR	NR
SCODI (25, 35)	?/Moderate	?/Moderate	+/High	NR	−/Low
SCPI (26, 27)	?/Moderate	−/High	+/High	?/Very low	−/High
DSMS (28)	?/Low	−/High	+/High	NR	−/Low
CDSMS (29)	?/Low	+/High	−/High	NR	−/High
SMOD-CA (30)	?/Low	?/Moderate	+/High	?/Very low	−/Low
C-SMOD-A-23 (31)	?/Low	+/High	+/High	+/Very low	−/Low
DSMI-20 (32)	?/Low	?/Moderate	+/High	?/Very low	NR
SDSCA (33)	?/Low	+/High	+/High	?/Very low	−/Low
SCI-R (34)	NR	−/High	+/High	NR	NR
DSMQ-R (22, 23)	NR	?/High	+/Low	NR	C
DSMQ (24)	NR	NR	NR	NR	C
SCODI (25, 35)	NR	NR	NR	NR	B
SCPI (27)	?/Low	?/Low	NR	NR	C
DSMS (28)	NR	NR	NR	NR	C
CDSMS (29)	NR	NR	NR	NR	C
SMOD-CA (30)	NR	NR	NR	NR	B
C-SMOD-A-23 (31)	NR	NR	NR	NR	B
DSMI-20 (32)	NR	?/Moderate	NR	NR	B
SDSCA (33)	NR	NR	NR	NR	B
SCI-R (34)	NR	NR	NR	NR	C

This table summarizes instrument-level evidence across included studies. For each measurement property, the symbol before the slash indicates the overall rating of the measurement property result, and the term after the slash indicates the quality of evidence. Measurement property results were rated as + = sufficient; − = insufficient; ? = indeterminate; NR = not reported. Evidence quality was graded as high, moderate, low, or very low according to the modified grading approach recommended by COSMIN. Recommendation levels were defined as follows: Grade A = instruments with sufficient content validity and at least low-quality evidence for sufficient internal consistency; Grade B = instruments that may be used with caution but require further validation; Grade C = instruments for which high-quality evidence indicates insufficient performance for at least one measurement property.

#### 3.3.1 Instrument development

Five studies (25–28, 35) clearly described the conceptual framework and theoretical model of the measurement instruments during instrument development. However, two studies focusing on cultural adaptation (26, 35) offered inadequate details of the cognitive interview procedure, resulting in a methodological quality rating of doubtful. The other three developmental studies (25, 27, 28) did not adopt appropriate qualitative methods for item generation during instrument design, resulting in a rating of doubtful. The remaining nine studies (22–24, 29–34) either provided limited information on the conceptual framework and instrument design or gave unclear accounts of cognitive interviews and pilot testing, and were thus rated as doubtful.

#### 3.3.2 Validity

##### 3.3.2.1 Content validity

Two revision studies of existing instruments (23, 34) did not report the instrument development process in terms of relevance, comprehensibility, and comprehensiveness, resulting in a methodological quality rating of not reported. The other 12 studies (22, 24–33, 35) reported content validity based on evaluations of all items by patients or experts. However, these studies showed insufficient methodological reporting, including the exclusive use of quantitative methods, insufficient descriptions of interviewers, interview procedures, and interview guides, and uncertainty about whether data were analyzed independently by two or more investigators. Consequently, the methodological quality was rated as doubtful or not reported, and the measurement property was classified as indeterminate.

##### 3.3.2.2 Structural validity

Nine studies (22–24, 26, 28, 29, 31, 33, 34) conducted both exploratory and confirmatory factor analyses with adequate sample sizes, resulting in a methodological quality rating of very good. Five studies (25, 27, 30, 32, 35) conducted exploratory factor analysis. Among them, one study (27) had a sample size less than five times the number of items, yielding a methodological quality rating of inadequate; the remaining studies had sufficient sample sizes and were rated as adequate. One study (25) used exploratory structural equation modeling and reported that all fit indices met the required criteria, indicating that the measurement properties were sufficient. The other four studies (27, 30, 32, 35) were classified as indeterminate. Ten studies (22–26, 28, 29, 31, 33, 34) reported comparative fit indices, of which six (22, 23, 25, 29, 31, 33) were above 0.95. Nine studies (22–26, 28, 29, 33, 34) reported root mean square error of approximation, of which five (22, 23, 25, 29, 33) were below 0.06. Five studies (23, 25, 28, 31, 33) reported standardized root mean square residual, of which four (23, 25, 31, 33) were below 0.08. Accordingly, the structural validity results of six studies (22, 23, 25, 29, 31, 33) were rated as sufficient. Four studies (24, 26, 28, 34) had comparative fit indices below 0.95, and their structural validity results were rated as insufficient.

##### 3.3.2.3 Criterion validity

Eight studies (23, 26, 28–31, 33, 35) reported results for criterion validity. Among them, five studies (28, 30, 31, 33, 35) used a reference standard other than the original scale, resulting in methodological quality and measurement property ratings of inadequate and insufficient, respectively. Three studies (23, 26, 29) employed the original well-established scale as the reference instrument, yielding a methodological quality rating of very good. However, their correlation coefficients with the reference standard were less than 0.7, so the measurement property was rated as insufficient. The remaining six studies (22, 24, 25, 27, 32, 34) did not report criterion validity.

##### 3.3.2.4 Hypothesis testing for construct validity

Five studies (22, 23, 26, 27, 32) conducted hypothesis testing to assess construct validity. Two studies (26, 27) conducted hypothesis testing via convergent validity and discriminant validity. One study (27) did not clearly describe subgroup characteristics, leading to a methodological quality rating of doubtful and a measurement property rating of indeterminate. The other study (26) provided complete and clear descriptions, with a methodological quality rating of adequate and a measurement property rating of sufficient. Two studies (23, 32) verified hypotheses through convergent validity and fully reported the structure and measurement properties of the reference instruments, resulting in a methodological quality rating of very good for both. One study (32) did not specify predefined hypotheses and was rated indeterminate for measurement property, while the other was rated sufficient. One study (22) tested for discriminant validity but did not clearly describe subgroup characteristics, resulting in a methodological quality rating of doubtful and a measurement property rating of indeterminate. The remaining nine studies (24, 25, 28–31, 33–35) did not report hypothesis testing.

##### 3.3.2.5 Measurement error and responsiveness

Among the 14 included studies, only one study (26) reported measurement error. However, it had deficiencies in statistical methodology, leading to a methodological quality rating of doubtful and a psychometric property rating of indeterminate. Only one study (23) reported responsiveness. The authors did not calculate correlations between change scores, resulting in a methodological quality rating of doubtful. The results were consistent with the predefined hypotheses, and the measurement properties were rated as sufficient.

#### 3.3.3 Reliability

##### 3.3.3.1 Internal consistency

All 14 studies assessed the internal consistency of the measurement instruments’ dimensions, and all received a methodological quality rating of very good. Nine studies (25–28, 30–33, 35) reported Cronbach’s *α* coefficients higher than 0.7 for all dimensions, so their psychometric property was rated as sufficient. Five studies (22–24, 29, 34) reported Cronbach’s α coefficients below 0.7 in some dimensions, resulting in a psychometric property rating of insufficient.

##### 3.3.3.2 Test–retest reliability

Eight studies (22, 23, 26, 27, 30–33) reported test–retest reliability. Two studies (22, 31) assumed stable patient composition, appropriate test–retest intervals, and consistent measurement settings and procedures, and calculated intraclass correlation coefficients (ICCs), resulting in an adequate methodological quality rating. Their ICC values exceeded 0.7, indicating that the psychometric property was sufficient. Three studies (26, 30, 32) did not calculate ICC but adopted Pearson correlation coefficients, yielding a doubtful methodological quality rating and an indeterminate rating for this psychometric property. Three studies (23, 27, 33) had inappropriate retest intervals, unstable patient composition, and did not report ICCs. They were therefore rated as inadequate in methodological quality and as indeterminate for this measurement property.

### 3.4 Evidence level and recommendations for included assessment instruments

#### 3.4.1 Risk of bias

All instruments, including DSMQ-R, DSMQ, SCODI, SCPI, DSMS, CDSMS, SMOD-CA, C-SMOD-A-23, DSMI-20, and SDSCA, received a methodological quality rating of doubtful for content validity and were therefore downgraded by one level. For structural validity, no downgrading was applied to DSMQ-R, DSMQ, SCPI, DSMS, CDSMS, C-SMOD-A-23, SDSCA, and SCI-R, as they were supported by multiple studies rated as adequate or by one study rated as very good. SCODI, SMOD-CA, and DSMI-20 were downgraded due to multiple studies rated as doubtful or only one study rated as adequate. For criterion validity, no downgrading was applied to DSMQ-R, SCPI, and CDSMS because one study achieved very good methodological quality. SCODI, DSMS, SMOD-CA, C-SMOD-A-23, and SDSCA were downgraded by 2 levels owing to multiple studies rated as inadequate or only 1 study rated as doubtful. For hypothesis testing, no downgrading was applied to DSMQ-R (two studies rated adequate). SCPI was downgraded by one level (one study rated adequate). DSMI-20 was downgraded by 1 level (only 1 study was rated adequate). For measurement error, SCPI was downgraded by 2 levels (only 1 study was rated as doubtful). For responsiveness, DSMQ-R was downgraded by 2 levels (only 1 study was rated inadequate). For internal consistency, all instruments were rated very good; thus, no downgrading was conducted.

For test–retest reliability, DSMQ-R and C-SMOD-A-23 were downgraded by 1 level (1 study rated adequate). SCPI, SMOD-CA, and DSMI-20 were downgraded by 2 levels (1 study rated doubtful). SDSCA was downgraded by 2 levels (only 1 study was rated inadequate).

#### 3.4.2 Inconsistency

DSMQ-R showed substantial inconsistency across studies in test–retest reliability and was downgraded by 2 levels. SCPI demonstrated serious inconsistency in structural validity (downgraded by 2 levels) and in test–retest reliability and hypothesis testing (downgraded by 1 level). SCODI showed consistent results across studies and was not downgraded. All other instruments were supported by only one independent study; therefore, no downgrading was applied for any measurement property.

#### 3.4.3 Imprecision

According to the COSMIN guidelines, DSMQ-R was not downgraded for test–retest reliability, as the cumulative test–retest sample size across two studies (22, 23) exceeded 100 participants. SCPI was downgraded by one level, with a cumulative test–retest sample size of 93 participants across two studies (26, 27). SMOD-CA, C-SMOD-A-23, DSMI-20, and SDSCA were downgraded by 2 levels, as their test–retest sample sizes were all fewer than 50 participants.

#### 3.4.4 Indirectness

All included instruments were validated within the target population of patients with diabetes, without extension to other groups, and showed no indirectness. Consequently, no downgrading was performed for the indirectness domain.

#### 3.4.5 Overall recommendations

Among the instruments assessing behavior-related diabetes self-management, six instruments (DSMQ-R, DSMQ, SCPI, DSMS, CDSMS, and SCI-R) were classified as Grade C because high-quality evidence indicated insufficient performance for at least one measurement property. The remaining five instruments (SCODI, SMOD-CA, C-SMOD-A-23, DSMI-20, and SDSCA) were classified as Grade B. Although these instruments showed sufficient internal consistency, their content validity was rated as indeterminate. It lacked sufficient or any evidence for criterion validity, cross-cultural validity, measurement error, and responsiveness. Therefore, Grade B instruments should be used cautiously and require further validation. A concise overview of the target populations, key measurement properties, and COSMIN recommendation levels of the included instruments is provided in Table 4.

**Table 4:** Summary of key measurement properties and COSMIN recommendation levels of the included diabetes self-management instruments.

Instrument name	Target population	Number of domains/items	Content validity	Structural validity	Internal consistency	Test–retest reliability	Responsiveness	Recommendation grade
DSMQ-R (22, 23)	T1DM and T2DM	5/27	?	+	−	?	+	C
DSMQ (24)	DM	4/16	?	−	−	NR	NR	C
SCODI (25, 35)	T2DM	4/40	?	?	+	NR	NR	B
SCPI (26, 27)	T1DM and T2DM	3/25	?	−	+	?	NR	C
DSMS (28)	T2DM	3/22	?	−	+	NR	NR	C
CDSMS (29)	T2DM	5/13	?	+	−	NR	NR	C
SMOD-CA (30)	T1DM	4/30	?	?	+	?	NR	B
C-SMOD-A-23 (31)	T1DM	4/23	?	+	+	+	NR	B
DSMI-20 (32)	T2DM	4/20	?	?	+	?	NR	B
SDSCA (33)	T2DM	5/11	?	+	+	?	NR	B
SCI-R (34)	T2DM	1/13	NR	−	+	NR	NR	C

This summary table provides a concise comparison of the included instruments with respect to target population, number of domains/items, selected key measurement properties, responsiveness, and final COSMIN recommendation grade. Only key measurement properties most relevant to instrument selection are shown in this table; more detailed study-level and instrument-level results are presented in Tables 2, 3. Measurement property results were rated as + = sufficient; − = insufficient; ? = indeterminate; NR = not reported. Recommendation grades were assigned according to COSMIN guidance: Grade A = recommended; Grade B = potentially useful but requiring further validation; Grade C = not recommended as a first-choice instrument because of insufficient evidence for at least one important measurement property. T1DM = type 1 diabetes mellitus; T2DM = type 2 diabetes mellitus; DM = diabetes mellitus.

## 4 Discussion

### 4.1 Methodological and psychometric quality of behavior-related diabetes self-management assessment instruments needs further improvement

This study demonstrated considerable room for improvement in the methodological quality of the 11 included assessment instruments. Most studies only reported some properties, such as content validity, structural validity, internal consistency, test–retest reliability, and criterion validity, with low evidence quality. Content validity is the most important psychometric property of an assessment instrument (36). Although most instruments in this study evaluated content validity, deficiencies in methodological reporting were noted. Five studies (25–28, 35) described the conceptual framework and theoretical model. However, they failed to elaborate on the qualitative research design and data collection procedures, resulting in methodological quality ratings that were doubtful and indeterminate for these measurement properties. Two revision studies (23, 34) lacked a clear description of the instrument development process.

According to the COSMIN guidelines, establishing content validity should fully account for the perspectives of the target population. Qualitative methods should be used to thoroughly explore participants’ perceptions of the relevance, comprehensibility, and comprehensiveness of the scale items. Therefore, when developing new assessment instruments in the future, researchers should strictly follow the COSMIN guidelines and clearly document the study design to guarantee methodological rigor and scientific soundness. Meanwhile, standardized qualitative approaches should be adopted for item generation. Researchers are encouraged to combine quantitative and qualitative methods and provide complete reporting of the entire evaluation process.

Sample size adequacy is a key methodological indicator for evaluating structural validity (37). A rigorous structural validity assessment should first explore the scale’s factor structure using exploratory factor analysis and then confirm the hypothesized structure in an independent sample using confirmatory factor analysis, thereby improving the robustness and interpretability of the findings (38). In the included studies, 9 studies (22–24, 26, 28, 29, 31, 33, 34) performed both exploratory and confirmatory factor analysis with adequate sample sizes, thus receiving a methodological quality rating of “very good”. Five studies (25, 27, 30, 32, 35) conducted only exploratory factor analysis; among them, one study (27) was rated “inadequate” in methodological quality because its sample size was less than five times the number of items. Another study (25) used only exploratory structural equation modeling and was rated “sufficient” in measurement properties. This study indicates that external validation of diabetes self-management assessment instruments in China remains inadequate. Insufficient sample size may lead to unstable factor structures and a higher risk of biased results (39). Notably, most studies relied solely on EFA without performing CFA, making it difficult to test the hypothesized structure rigorously and leaving the scale framework without sufficient external support. This falls short of the validation standards used for internationally established scales such as DSMQ, DSMQ-R, and SDSCA.

In future research, sample size should be estimated scientifically based on scale dimensions and model specifications. Priority should be given to CFA or ESEM for validation, with standardized selection of factor analysis methods and complete reporting of fit indices. For scales with inadequate model fit, cross-sample validation, item revision, or exploration of alternative models should be considered.

Reliability refers to the degree to which scores are free from measurement error and includes test–retest reliability, inter-rater reliability, and intra-rater reliability. Since all included instruments were self-report questionnaires, the reliability evidence focused mainly on test–retest reliability (40). Among the included scales, eight studies (22, 23, 26, 27, 30–33) reported test–retest reliability, while only two studies (22, 31) were rated adequate in methodological quality. Three studies (23, 27, 33) adopted highly variable retest intervals, ranging from two weeks to 3–6 months. A short retest interval may cause recall bias, as participants remember their previous answers, thereby undermining the accuracy of the results. Conversely, an excessively long interval may increase participant dropout or trigger real behavioral changes influenced by external factors. Some studies have reported a negative correlation between retest interval and reliability, such that longer intervals are associated with lower reliability coefficients (41). Most studies did not clearly describe participant stability throughout the retest period or the consistency of measurement conditions. Several studies failed to justify their selection of retest intervals and presented unstable participant composition. In this review, the methodological quality ratings for certain test–retest reliability outcomes may have underestimated the actual test–retest reliability of these scales when used to assess self-management among individuals with diabetes. Future studies should standardize the design of test- retest protocols, clarify the rationale for interval selection, participant stability, and measurement conditions, and fully report ICC or weighted Kappa values to improve the quality of test–retest reliability evaluations.

Criterion validity reflects the degree of agreement between an assessment instrument and a “gold standard” (42). In this review, eight studies (23, 26, 28–31, 33, 35) reported criterion validity. Among them, five studies (22, 24, 25, 27, 29) used a reference standard other than the original scale. Although three studies (23, 26, 29) used well-established and valid original scales as criteria, the correlation coefficients between the newly developed instruments and the criteria were all below 0.7, indicating inadequate measurement properties. This finding indicates that, even with a rigorous study design, the scale’s measurement performance may lack sufficient agreement with the “gold standard,” hindering optimal criterion validity. A potential explanation is that diabetes self-management instruments assess multidimensional constructs, and there is currently no universally accepted gold-standard measurement tool, making it challenging for researchers to select an appropriate criterion. Lee suggested that, in the absence of a gold standard, instruments that are similar in their measured structure or are multidimensional and comprehensive can serve as a standard (43). Future research should aim to establish consensus on the criteria. It is recommended to prioritize 1–2 universally accepted scales as the preferred gold standard or to select instruments with the most conceptually consistent structure as the gold standard.

Additionally, researchers may calculate indicators such as the Area Under the Curve (AUC), sensitivity, and specificity to determine optimal cut-off values for each level. Given the absence of a universal gold standard for diabetes self-management behaviors, evidence presented as criterion validity in the included studies should be interpreted with caution. In some scenarios, such evidence may be more appropriately categorized as construct validity. When using revised scales, their measurement properties should be fully validated and reported in advance.

### 4.2 The reporting of measurement properties among diabetes self-management assessment instruments remains incomplete

None of the 14 included studies reported the cross-cultural validity of the assessment instruments. Only one study assessed measurement error (26), and another reported responsiveness (23). The lack of cross-cultural validity means these instruments have not been shown to achieve measurement equivalence across languages, cultures, and regional contexts, limiting their use in cross-national, cross-ethnic, and multicenter comparative research. Cultural backgrounds, dietary habits, health beliefs, and other factors exert profound impacts on diabetes self-management behaviors. Insufficient cross-cultural validation may lead to misinterpretation of scale items and reduce reliability and validity, further limiting their broad application. Therefore, researchers are advised to fully consider the cultural context of the target population during instrument development and conduct cross-cultural evaluations in the validation stage to compare performance across diverse cultural settings. This can help clinical nurses accurately assess self-management status and formulate targeted interventions.

Evidence for measurement error, responsiveness, and cross-cultural validity was largely absent. Measurement error reflects the extent to which observed scores deviate from true scores, whereas responsiveness indicates an instrument’s ability to detect meaningful changes over time (44). Without evidence for these properties, it is difficult to determine whether observed score changes reflect true improvements in self-management behaviors or are attributable to random error, systematic bias, or changes in measurement conditions.

The absence of evidence of responsiveness is particularly important for intervention studies and longitudinal follow-up. Although some instruments may be suitable for cross-sectional descriptive assessment or preliminary screening, they cannot yet be strongly recommended as core outcome measures for evaluating diabetes education, behavioral interventions, digital health programs, or nursing interventions.

Cross-cultural validity was also not reported in the included studies. This means that measurement equivalence across languages, cultures, or regions has not been established. Given that diabetes self-management behaviors are strongly influenced by cultural background, dietary habits, healthcare access, and health beliefs, future studies should evaluate cross-cultural validity using methods such as multi-group confirmatory factor analysis or differential item functioning analysis (45).

However, only one study (23) in this review reported responsiveness, yielding inadequate evidence to support most instruments for evaluating longitudinal intervention effects. Accordingly, although several scales can be used for cross-sectional assessment or clinical screening, none of the included tools can be strongly recommended for long-term follow-up or for evaluating intervention outcomes. These deficiencies in measurement properties directly lower the confidence of the final recommendation grades. In accordance with the COSMIN criteria, Grade B instruments do not receive strong endorsement; instead, they require cautious use given the current evidence and call for further validation. In other words, while Grade B tools such as SDSCA and C-SMOD-A-23 show acceptable applicability in specific populations and scenarios, their recommendations remain tentative. Especially in the absence of evidence on cross-cultural validity, measurement error, and responsiveness, these instruments are more appropriate for descriptive assessment, preliminary screening, and cross-sectional studies. They are not yet suitable as core outcome measures for cross-cultural comparisons or for evaluating intervention effectiveness.

Only five studies (22, 23, 26, 27, 32) conducted hypothesis testing. The primary reason is that the other nine studies focused on clinical applications and basic reliability and validity tests, mainly reporting fundamental indicators such as Cronbach’s *α* and factor analysis, without developing relevant theoretical hypotheses. Hypothesis testing is widely adopted to verify established theoretical structures. In contrast, some locally developed scales are still in the early stages of development and lack fully developed theoretical frameworks, making it challenging to formulate explicit research hypotheses.

Future studies should standardize reporting of measurement properties, strengthen the applicability and measurement precision of instruments, and place greater emphasis on theoretical hypothesis development. These efforts will facilitate the cross-cultural and cross-linguistic application of these tools and comprehensively improve their methodological quality and clinical utility.

### 4.3 SDSCA and C-SMOD-A-23 are tentatively recommended but require further improvement and validation

The findings of this study indicate that none of the available instruments for assessing behavior-related self-management in diabetes met the COSMIN criteria for grade A recommendation. Although the SDSCA and C-SMOD-A-23 were classified as Grade B and demonstrated acceptable applicability in their respective target populations, both were limited by indeterminate content validity, inadequate criterion validity, and a complete lack of evidence for cross-cultural validity, measurement error, and responsiveness. Accordingly, these two instruments are considered provisional alternatives, requiring cautious application and use under the current evidence base, rather than strongly recommended tools with fully confirmed psychometric performance.

For adults with type 2 diabetes mellitus, the Chinese version of the SDSCA serves as a relatively clinically feasible alternative. This scale covers core self-management domains specific to type 2 diabetes, including dietary control, regular physical activity, blood glucose monitoring, foot care, and medication adherence. Its items are tailored to the behavioral features of adults with type 2 diabetes, a group whose management mainly relies on lifestyle modification and long-term chronic disease care, with no items targeting type 1 diabetes-specific content, such as insulin dose adjustment or acute hypoglycemia management. Its domain structure aligns closely with the self-management demands of this population. The SDSCA was rated sufficient for construct validity and internal consistency, indicating adequate empirical support for its factor structure and item homogeneity across available studies.

Nevertheless, its content validity remains indeterminate; available evidence for test–retest reliability is limited; criterion validity is inadequate; and data on measurement error and responsiveness are absent. Compared with generic diabetes scales that lack a targeted design for type 2 diabetes behaviors, the SDSCA presents superior population suitability and clinical utility. Other included instruments (e.g., DSMQ, SCI-R) are predominantly generic tools with poor specificity and insufficient emphasis on core lifestyle self-management behaviors of type 2 diabetes patients. Meanwhile, the SDSCA features concise, easy-to-comprehend items, imposes minimal response burden and short administration time, rendering it well-suited for rapid clinical screening, regular follow-up assessments, and community epidemiological investigations. Given its extensive use among Chinese adults with type 2 diabetes and its practical feasibility, the SDSCA may be prioritized for brief clinical or community-based assessment in this population, provided that its evidence limitations are acknowledged. However, this conclusion should not be directly extended to older adults with diabetes. The available SDSCA validation evidence was not generated specifically in older populations, and its suitability for older people with frailty, cognitive impairment, functional limitations, sensory decline, or caregiver-supported self-management remains uncertain (8–10). In particular, the SDSCA focuses mainly on individual behavioral frequency over the previous seven days and may not fully capture shared responsibility, caregiver assistance, treatment simplification, or safety-oriented self-management priorities that are highly relevant to older adults.

For adolescents with type 1 diabetes mellitus, the C-SMOD-A-23 demonstrates strong population specificity. This scale centers on daily care activities, problem-solving, parent–adolescent collaboration, diabetes-related communication, and goal setting relevant to type 1 diabetes management among adolescents, capturing the contextual characteristics of self-management in this population. It was purposefully developed for adolescents with type 1 diabetes to assess their self-management behaviors, with items closely aligned with their practical daily needs. This review shows that the C-SMOD-A-23 was rated as sufficient for construct validity, internal consistency, and test–retest reliability, suggesting a solid, reliable measurement basis for adolescent populations with type 1 diabetes. However, its content validity remains indeterminate, criterion validity is inadequate, and no supporting evidence exists for cross-cultural validity, measurement error, or responsiveness. This instrument fills the gap in specialized assessment instruments for type 1 diabetes and possesses promising clinical utility. Other included scales (e.g., DSMQ, SCI-R) are predominantly generic or developed for type 2 diabetes and cannot capture core behaviors specific to type 1 diabetes, such as insulin dose adjustment and acute hypoglycemia management. Written in straightforward wording and requiring brief administration time, this scale may be useful for routine clinical follow-up and descriptive assessment in adolescents with type 1 diabetes; however, further evidence on responsiveness and measurement error is needed before it can be confidently used to evaluate intervention effects.

Overall, the primary strengths of SDSCA and C-SMOD-A-23 include clearly specified target populations, items closely aligned with routine diabetes self-management behaviors, and acceptable evidence for several core measurement properties. Their major drawbacks include indeterminate content validity, inadequate criterion validity, and the absence of evidence for longitudinal psychometric indicators. Accordingly, clinicians and researchers need to comprehensively consider the target population, assessment objectives, time required, and limitations in the existing evidence when choosing an instrument for practical use.

### 4.4 Implications for self-management assessment in older adults with diabetes

Although this review included instruments validated in patients with diabetes across different age groups, the current evidence base provides limited support for their direct application to older adults with diabetes. None of the included studies specifically focused on older adults as the primary target population, and no validation study examined whether instrument performance differed according to frailty, multimorbidity, cognitive impairment, functional decline, sensory limitations, polypharmacy, or caregiver dependence. This represents an important evidence gap in the context of population ageing and the increasing burden of diabetes among older people (2, 7).

Self-management in older adults with diabetes may differ substantially from that in younger and middle-aged adults (8, 9). Frailty and functional decline may limit the ability to engage in regular physical activity, prepare appropriate meals, perform foot care, or attend follow-up visits (8–10). Cognitive impairment may affect medication adherence, insulin use, recognition of hypo- or hyperglycemia, and the accuracy of self-reported behaviors (8, 11). Multimorbidity and polypharmacy may complicate treatment priorities and make diabetes-specific self-management only one component of a broader chronic disease management burden (8, 9, 12). Sensory limitations, such as impaired vision or hearing, may reduce the comprehensibility of written questionnaire items and the feasibility of independent questionnaire completion (8, 9). In addition, many older adults rely on spouses, adult children, or formal caregivers for medication management, dietary preparation, blood glucose monitoring, and healthcare access. Therefore, instruments that assume fully independent self-management may underestimate or misclassify self-management capacity in this population.

These age-related characteristics have direct implications for content validity. Existing instruments commonly assess behaviors such as diet, physical activity, glucose monitoring, medication adherence, and foot care. However, they may not adequately capture caregiver-supported behaviors, shared responsibility, treatment simplification, hypoglycemia prevention, functional feasibility, or individualized glycemic goals in older adults. Furthermore, instruments with longer item sets, complex response formats, or short recall periods may impose additional response burden on older adults with cognitive or sensory impairment. Shorter instruments such as the SDSCA may be feasible in busy clinical settings, but feasibility alone does not establish validity for older adults. Its content validity, test–retest reliability, measurement error, and responsiveness in older populations remain insufficiently established.

The lack of age-specific validation also limits score interpretation. Without evidence of measurement invariance across age groups, it remains unclear whether older and younger adults interpret items in the same way, or whether observed score differences reflect true differences in self-management behavior rather than age-related differences in cognition, function, health literacy, or caregiver involvement. Similarly, the absence of evidence for measurement error and responsiveness means that existing instruments cannot yet be confidently used to evaluate changes in self-management among older adults following diabetes education, geriatric assessment, medication review, caregiver training, or community-based interventions.

Future validation studies should therefore prioritize older adults with diabetes, especially those with frailty, multimorbidity, cognitive impairment, functional limitations, sensory impairment, polypharmacy, and caregiver dependence (8–12). Content validity studies should include not only older patients but also caregivers and healthcare professionals involved in geriatric diabetes care (8–10, 13). Cognitive interviews should be used to determine whether items are relevant, comprehensible, and comprehensive for older adults with different levels of cognitive and functional ability (13). Psychometric testing should further examine age-stratified structural validity, reliability, measurement error, responsiveness, and measurement invariance (13, 44, 45). Where necessary, existing instruments may need to be revised or supplemented to incorporate caregiver-assisted self-management, treatment burden, safety-oriented behaviors, and individualized goals that reflect the realities of diabetes care in later life.

### 4.5 Applicability of different instruments in clinical and research settings

Instrument selection depends not only on psychometric quality but also on the specific context of use. In clinical settings—particularly outpatient clinics, primary care facilities, and community follow-ups—assessment time is limited, and patient turnover is high; therefore, brevity, comprehensibility, ease of scoring, and low response burden are particularly important. From this perspective, shorter instruments such as SDSCA, DSMI-20, CDSMS, and SCI-R deliver practical operational advantages. The SDSCA consists of only 11 items with a 7-day recall frequency, enabling rapid evaluation of recent self-management behaviors in diet, exercise, blood glucose monitoring, and foot care, making it highly appropriate for brief outpatient screening and community surveys. The DSMI-20 comprises 20 items, and its original validation reported an average completion time of around five minutes, indicating satisfactory clinical feasibility. Nevertheless, clinical feasibility does not equal adequate psychometric quality. For instance, although DSMI-20 was rated sufficient for internal consistency, evidence regarding construct validity and test–retest reliability remains inadequate or indeterminate, mandating further validation work.

In research settings—especially intervention trials, longitudinal follow-up studies, and theoretical model verification—selected instruments need acceptable internal consistency and construct validity, alongside robust evidence of measurement error and responsiveness. The SCODI is built on an explicit theoretical framework and covers domains of self-care maintenance, monitoring, management, and self-efficacy, making it well-suited for theory-driven, comprehensive research; however, its lengthy item set may increase participant burden. The DSMQ-R addresses dietary behavior, medication adherence, blood glucose monitoring, physical activity, and collaboration with healthcare providers and has been tested across large samples, supporting its application in exploratory or multidimensional behavioral assessments. Nonetheless, it was assigned Grade C overall, with inadequate evidence for internal consistency, criterion validity, and test–retest reliability, meaning it cannot serve as a primary outcome instrument at present. C-SMOD-A-23 is preferable for research targeting adolescents with type 1 diabetes, yet still needs supplementary evidence on responsiveness and measurement error. In geriatric diabetes care, instrument selection should also consider whether the assessed behaviors are realistic and clinically meaningful for older adults. For example, strict dietary control, independent glucose monitoring, regular exercise, and complex medication management may be difficult for older adults with frailty, multimorbidity, cognitive impairment, or functional decline. In such cases, assessment should not focus only on whether patients independently perform self-management behaviors, but should also consider whether appropriate support systems, caregiver involvement, simplified treatment regimens, and safety-oriented behaviors are in place. At present, the included instruments provide insufficient evidence to determine whether they can capture these geriatric-specific aspects of diabetes self-management.

Since nearly all included instruments lacked reporting of measurement error and responsiveness, it is hard to judge whether observed score changes correspond to genuine improvements in self-management or stem from random error, systematic bias, or inconsistent testing conditions. Accordingly, based on available evidence, most tools are appropriate merely for cross-sectional descriptive assessments. They are not recommended as standalone measures to quantify long-term outcomes of diabetes education, behavioral interventions, digital health initiatives, or nursing-based interventions.

Furthermore, none of the 14 included studies reported cross-cultural validity or measurement invariance. Hence, even for translated instrument versions, the equivalence of item interpretation and factor structure across diverse cultural groups cannot be verified. Prior to application in cross-cultural comparative research, researchers should test measurement equivalence via multi-group confirmatory factor analysis or differential item functioning analysis.

### 4.6 Practical administration burden of different instruments

Practical administration burden is a key factor influencing the clinical implementation of assessment instruments, covering item quantity, domain structure, response format, scoring complexity, and completion time (46). However, this review found that most included studies failed to report scale completion time. Therefore, the actual administration burden could be inferred only indirectly from item counts, response formats, and scoring procedures. For older adults, administration burden should be interpreted more broadly than item number alone. Visual impairment, hearing loss, cognitive decline, fatigue, low health literacy, and the need for caregiver assistance may all affect the feasibility and accuracy of self-report completion (8, 9, 11). Therefore, an instrument that appears brief and simple in general adult populations may still be difficult to administer or interpret in older adults with functional or cognitive limitations (8, 9).

The SDSCA imposes the lowest administration burden. It consists of 11 items with a 7-day frequency recall format, featuring concise item wording and straightforward scoring, which renders it ideal for rapid outpatient screening and community diabetes management (47). The CDSMS and SCI-R each contain 13 items and theoretically carry low administration burden; nevertheless, both were classified as Grade C with inadequate evidence for construct validity, internal consistency, and content validity. Accordingly, they cannot be prioritized solely on account of their shorter length. DSMI-20 and C-SMOD-A-23 fall into the moderate-burden category. The DSMI-20 comprises 20 items, and its original validation reported an average completion time of approximately five minutes, fitting clinical and community scenarios with restricted time but a need for multidimensional evaluation. The C-SMOD-A-23 has 23 items and requires around 12–15 min for administration, making it more appropriate for specialized adolescent type 1 diabetes clinics, structured education interventions, or research environments. By contrast, it proves too time-intensive for high-volume outpatient services. The SMOD-CA includes 30 items and can be finished within 20 min for a comprehensive assessment among adults with type 1 diabetes, yet it lacks convenience for routine outpatient use.

The DSMQ-R and SCODI achieve comprehensive content coverage but carry a relatively high administration burden. The DSMQ-R contains 20 core items and 7 optional items, and the full-scale administration adds extra workload for scoring and result interpretation. With 40 items, the SCODI has the largest item bank across all included instruments. Despite its solid theoretical grounding and extensive content coverage, its lengthy item set limits routine clinical application. Hence, the SCODI is more recommended for research or full-scale assessment than for brief outpatient screening.

In summary, instrument selection requires a balance between psychometric quality and practical feasibility. Shorter scales are preferred for quick clinical screening at the potential cost of incomplete content coverage. In contrast, longer instruments are better suited to research and comprehensive evaluation but require robust evidence on validity, responsiveness, and measurement error to justify their clinical application (48). In ageing populations, feasibility should therefore be evaluated together with accessibility and interpretability (8, 9, 13). Future studies should report completion time, missing response rates, need for assistance, and acceptability among older adults with different levels of cognitive and functional ability (13). Such evidence is necessary before existing instruments can be confidently recommended for routine geriatric diabetes care.

### 4.7 Interpretation of COSMIN recommendation levels and implications for instrument selection

Within the COSMIN recommendation framework, Grade B does not constitute a strong endorsement for an instrument. Instead, it indicates that the existing evidence is insufficient to categorize the tool as either Grade A or Grade C, suggesting cautious clinical application and a pressing need for further validation. Grade C is assigned when high-quality evidence confirms inadequate performance for at least one core psychometric property; such scales are generally not recommended as first-choice unless superior alternative measures are unavailable. Accordingly, despite the respective suitability of SDSCA for adults with type 2 diabetes and C-SMOD-A-23 for adolescents with type 1 diabetes, their application still requires caution. Notably, their content validity remains indeterminate, and consistent evidence for cross-cultural validity, measurement error, and responsiveness is absent or inadequate across available studies.

The findings of this review also reveal that poor methodological quality directly downgrades the final recommendation grade of assessment instruments. Inadequate documentation of qualitative procedures during content validity assessment constitutes a major barrier preventing scales from attaining higher recommendation grades. Even several instruments with acceptable construct validity or internal consistency failed to reach Grade A owing to insufficient rigor in reporting content validity evidence. Some investigations conducted only exploratory factor analysis without follow-up confirmatory factor analysis in independent samples, thereby compromising the robustness of construct validity evidence. Regarding test–retest reliability assessments, multiple studies adopted inappropriate retest intervals, failed to verify participant stability across the testing period, or used Pearson correlation coefficients rather than intraclass correlation coefficients (ICCs), all of which reduced the overall quality of the supporting evidence. For criterion validity, a universally accepted gold standard is lacking; several studies used poorly validated, adapted scales as reference criteria, further degrading the quality of the evidence. Therefore, the recommendation grades derived from this review mainly reflect the completeness and quality of existing evidence rather than a definitive judgment on the inherent superiority or inferiority of each instrument.

Future research needs improvement across multiple domains. During instrument development or cross-cultural adaptation, content validity testing must strictly comply with COSMIN guidelines, which encompass cognitive interviews with target participants and systematic appraisal of item relevance, comprehensibility, and comprehensiveness. Researchers should fully report interview protocols, interviewer information, verbatim transcripts, coding procedures, and data saturation outcomes. For construct validity analyses, confirmatory factor analysis or exploratory structural equation modeling should be performed with adequate sample sizes, and complete reporting of model fit indices, including CFI, TLI, RMSEA, and SRMR, should be provided. For test–retest reliability, investigators need to select rational retest intervals, confirm stable participant health and self-management status throughout the interval, and prioritize ICCs with corresponding confidence intervals over reliance on Pearson correlation coefficients alone. More studies targeting measurement error and responsiveness are required, with complete reporting of the standard error of measurement, minimal detectable change, minimal important difference, and area under the curve to justify the applicability of instruments for longitudinal follow-up and intervention outcome evaluation. Prior to cross-linguistic, cross-regional, and cross-cultural implementation, cross-cultural validity and measurement invariance should be tested to ensure valid intergroup comparisons. The interpretation of recommendation grades should also consider indirectness related to age-specific applicability. Although the included instruments were validated in patients with diabetes, most validation samples were not specifically composed of older adults, and age-related factors were rarely considered in study design or analysis. Therefore, the absence of COSMIN downgrading for indirectness in the general evidence synthesis should not be interpreted as evidence that these instruments are fully applicable to older adults with diabetes. Rather, the lack of age-specific validation should be recognized as a separate and clinically important limitation.

In summary, the overall evidence base for existing behavioral diabetes self-management instruments remains inadequate. Clinicians and researchers ought to select assessment instruments after an integrated consideration of diabetes subtype, target population, research objective, psychometric performance, and practical administration burden, rather than making choices based solely on superficial indicators such as short item length, favorable internal consistency, or widespread popularity.

## 5 Limitations

This study has several limitations: ① Only Chinese and English literature were included, which may limit the comprehensiveness of the evaluation of available instruments. ② Some COSMIN criteria involve subjective judgment, which may introduce potential bias. ③ Although this review assessed the major COSMIN measurement properties, many included studies did not report several key properties, which limited the completeness of the evidence synthesis. ④ Due to substantial heterogeneity across included studies in construct definition, sample sources, statistical methods, scale versions, and reported indicators, this study adopted the narrative synthesis approach recommended by the COSMIN group rather than performing quantitative meta-analysis. ⑤ Age-specific applicability could not be fully evaluated because most included studies did not target older adults or report measurement properties separately for older age groups. As a result, the suitability of existing instruments for older adults with frailty, multimorbidity, cognitive impairment, functional decline, sensory limitations, polypharmacy, or caregiver dependence remains uncertain. Therefore, comparisons of differences between different instruments should be interpreted with caution. Future research should strengthen training for researchers on the COSMIN standards to reduce rating bias. In addition, full reporting of all measurement properties is encouraged to identify current methodological gaps better and inform the development of improved scales.

## 6 Conclusion

This systematic review was conducted according to the COSMIN guidelines to assess 11 behavior-related diabetes self-management instruments, based on 14 eligible Chinese and English studies. The evaluation encompassed core measurement properties: content validity, structural validity, internal consistency, test–retest reliability, criterion validity, measurement error, responsiveness, and hypothesis testing. Results showed that five of the 11 instruments were graded as Grade B and six as Grade C. Among these options, SDSCA can be tentatively applied to adults with type 2 diabetes mellitus, given its adequate structural validity and internal consistency, as well as satisfactory feasibility for brief clinical and community-based screening. C-SMOD-A-23 covers self-management domains tailored to adolescents with type 1 diabetes. It provides relatively favorable evidence for structural validity, internal consistency, and test–retest reliability, though its overall evidence base remains incomplete. This instrument fills a gap in specialized assessment scales for type 1 diabetes and is therefore more appropriate for adolescent patients with this condition.

Future development and validation of self-management instruments should strictly comply with COSMIN standards and fully report all measurement properties, with particular focus on cross-cultural validity, measurement error, and responsiveness. Additional requirements include rigorous sample-size calculations, standardized factor-analytic procedures, and well-designed hypothesis testing. Moreover, more high-quality, disease-specific tools targeting type 1 and type 2 diabetes populations need to be developed to enable accurate clinical evaluation of diabetes self-management and support nurses in developing targeted, individualized intervention strategies. In the context of population ageing, particular attention should be paid to the lack of validation evidence for older adults with diabetes. Existing instruments have not been adequately tested in older populations characterized by frailty, multimorbidity, cognitive impairment, functional decline, sensory limitations, polypharmacy, and caregiver-supported self-management. Future instrument development and validation should explicitly include older adults and their caregivers, evaluate age-specific content validity and measurement invariance, and determine whether existing tools can validly capture the self-management behaviors, safety priorities, and support needs of ageing populations with diabetes.

## Data Availability

The original contributions presented in the study are included in the article/Supplementary material, further inquiries can be directed to the corresponding author.
