# Supplementary material for: Systematic review of behavior-related self-management assessment instruments for patients with diabetes
Source: Front Public Health. 2026 Aug 28;14:1858702. doi: 10.3389/fpubh.2026.1858702 (PMC13562226; doi:10.3389/fpubh.2026.1858702)
Supplement: Supplementary file 1 [file Data_Sheet_1.docx]

Supplementary Material

# 1 Supplementary Figures and Tables

## Supplementary Figures

**Supplementary Figure 1 PubMed Search Strategy.**

**Supplementary Figure 2 Chinese Biomedical Literature Service System Search Strategy**

**Supplementary Figure 3 EmBase Search Strategy**

**Supplementary Figure 4 China National Knowledge Infrastructure Search Strategy**

**Supplementary Figure 5 Wanfang Data Search Strategy**

**Supplementary Figure 6 VIP Chinese Journal Database Search Strategy**

**Supplementary Figure 7 Web of science** **Search Strategy**

**Supplementary Figure 8 CINAHL** **Search Strategy**

## Supplementary Tables

## Supplemental Table 1 COSMIN methodological quality ratings and study-level measurement property results of the included studies

| **Included studies** | **Scale Development and Evaluation** | **Content validity** | | | **Structural validity** | | **Internal consistency** | | **Test–retest reliability** | | **Criterion validity** |
| --- | --- | --- | --- | --- | --- | --- | --- | --- | --- | --- | --- |
|  |  | **Relevancen** | **Comprehensibility** | **Comprehensiveness** | **Indicator** | **COSMIN rating** | **Cronbach’s α coefficient** | **COSMIN rating** | **ICC/Kappa value** | **COSMIN rating** |  |
| Oliveira D(22) | D | D | D | NR | CFA: CFI=0.989, RMSEA=0.039 | V/+ | 0.62–0.97 | V/- | ICC: 0.96 | A/+ | NR |
| Schmitt A(23) | NR | NR | NR | NR | CFA: 5 factors;CFI=0.95-0.97, RMSEA=0.06-0.07 | V/+ | 0.84–0.92 | V/- | Test–retest reliability  : 0.53-0.64 | I/? | V/- |
| Li CQ(24) | D | D | D | D | EFA:5 factors; CFA: CFI=0.908, RMSEA=0.070 | V/- | 0.651–0.899 | V/- | NR | | NR |
| Ausili D(25) | D | D | NR | D | ESEM: ESEM:CFI=0.98-0.99, RMSEA=0.031-0.129 | A/+ | 0.81–0.89 | V/+ | NR | | NR |
| Wang X(26) | D | D | D | D | CFA:RMSEA=0.069, CFI=0.903 | A/- | 0.81–0.88 | V/+ | Test–retest reliability  : r=0.61 | D/? | V/- |
| Mbuagbaw L(27) | D | D | NR | D | EFA: 6 factors | I/? | 0.83–0.88 | V/+ | ICC: 0.48 | I/? | NR |
| Dai Z(28) | D | D | NR | NR | CFA: CFI=0.937, RMSEA=0.073 | V/- | 0.630–0.938 | V/+ | NR | | I/- |
| Şahin Yildiz Y(29) | D | D | D | NR | CFA: CFI=0.970, RMSEA=0.032 | V/+ | 0.407–0.785 | V/- | NR | | V/- |
| Zhu S(30) | D | D | NR | D | EFA: 4 factors | A/? | 0.773–0.857 | V/+ | Test–retest reliability: 0.816-0.925 | D/? | I/- |
| Lee S L(31) | D | D | NR | D | CFA: CFI=0.96, RMSEA=0.06 | V/+ | 0.70–0.88 | V/+ | ICC: 0.78–0.93 | A/+ | I/- |
| Lee C L(32) | D | D | D | D | EFA: 4 factors | A/- | 0.838–0.925 | V/+ | Test–retest reliability  : r=0.79 | D/? | NR |
| Hua L (33) | D | D | NR | NR | CFA: CFI=0.99, RMSEA=0.052 | V/+ | 0.829–0.913 | V/+ | Test–retest reliability  : 0.425-0.774 | I/? | I/- |
| Khagram L(34) | NR | NR | NR | NR | CFA: CFI=0.659-0.677, RMSEA=0.111-0.125 | V/- | 0.77 | V/+ | NR | | NR |
| Tang XR (35) | D | D | D | NR | EFA: 4 subscales, all item loadings > 0.4 | A/? | 0.709–0.908 | V/+ | NR | | I/- |

Note: This table presents the study-level COSMIN methodological quality ratings and measurement property results for the 14 included studies. For structural validity, internal consistency, test–retest reliability, and criterion validity, the first symbol in each COSMIN rating indicates the methodological quality of the study for that measurement property, and the second symbol indicates the rating of the measurement property result. Methodological quality was assessed using the COSMIN Risk of Bias checklist and rated as V = very good, A = adequate, D = doubtful, I = inadequate, and NR = not reported. Measurement property results were rated as + = sufficient, − = insufficient, and ? = indeterminate according to COSMIN criteria for good measurement properties. Relevance, comprehensibility, and comprehensiveness are the three components of content validity. CFA = confirmatory factor analysis; EFA = exploratory factor analysis; ESEM = exploratory structural equation modeling; CFI = comparative fit index; RMSEA = root mean square error of approximation; ICC = intraclass correlation coefficient; NR = not reported.

# 1.3 Supplementary PRISMA - COSMIN for OMIs 2024 Report List

| 1. **Title** | | | | |
| --- | --- | --- | --- | --- |
| **Item** | **Reporting requirement** | **Status** | **Reported information in this manuscript** | **Location / suggested page** |
| 1 | Identify the report as a systematic review and include the outcome domain, population, type/name of outcome measurement instruments, and measurement properties. | Reported | The title identifies the study as a systematic review of behavior-related self-management assessment instruments for patients with diabetes and reflects the target population, instrument type, and measurement focus. | Title page, p. 1 |
| 2. **Abstract** | | | | |
| 2.1 Objectives | State the main objective or question addressed by the review. | Reported | The abstract states that the review aimed to evaluate the methodological quality and measurement properties of behavior-related self-management assessment instruments for patients with diabetes. | Abstract – Objective, p1 |
| 2.2 Funding | State the main source of funding for the systematic review. | Not Reported | The review was supported by the Shanxi Health Commission Project (2025ZYYC011). | Funding statement, p16 |
| 2.3 Registration | Provide the registry name and registration number. | Reported | The abstract reports registration in PROSPERO under registration number CRD420251178394. | Abstract / Methods, p2 |
| 2.4 Background | Provide brief background and rationale. | Reported | The abstract and introduction summarize the importance of diabetes self-management and the need for validated behavior-related self-management instruments. | Abstract / Introduction, p1;p2 |
| 2.5Eligibility criteria | Summarize the inclusion and exclusion criteria. | Reported | The abstract briefly summarizes the target population, instrument construct, and requirement that at least one COSMIN measurement property be evaluated. | Abstract, p2 |
| 2.6 Information sources | Specify information sources and the date of last search. | Reported | The abstract reports the searched Chinese and English databases and the final search date of December 15, 2025. | Abstract, p1 |
| 2.7 Risk of bias | State the method used to assess risk of bias. | Reported | The abstract states that the COSMIN guideline/checklist was used to assess methodological quality. | Abstract, p1 |
| 2.8 Measurement properties | State how measurement property results were assessed. | Reported | Measurement properties were evaluated according to COSMIN criteria. | Abstract, p1 |
| 2.9 Synthesis methods | Summarize methods used to synthesize results. | Not Reported | Narrative synthesis and COSMIN-based evidence synthesis were used because of substantial heterogeneity across instruments and studies. | Methods / Limitations, p4, p12 |
| 2.10 Included studies | Provide the number of included studies and instruments. | Reported | Fourteen studies involving 11 assessment instruments were included. | Abstract, p1 |
| 2.11 Synthesis of results | Present synthesized results and certainty of evidence. | Reported | The abstract reports measurement property ratings, evidence levels, and recommendation grades. No Grade A instrument was identified; five were Grade B and six were Grade C. | Abstract, p1 |
| 2.12 Limitations of evidence | Summarize limitations of the evidence. | Reported | The abstract states that content validity evidence was often indeterminate and that cross-cultural validity, measurement error, and responsiveness were largely absent. | Abstract, p1 |
| 2.13 Interpretation | Provide a general interpretation of the findings. | Reported | The abstract concludes that current evidence remains insufficient and that SDSCA and C-SMOD-A-23 may be cautiously considered for specific populations. | Abstract, p1 |
| 3. **Plain language summary** | | | | |
| 3 | Provide a plain language summary if required by the journal. | Not applicable | A plain language summary was not required by the target journal. | Not applicable |
| 4. **Open science** | | | | |
| 4a Registration information | Provide the registry name and registration number, or state that the review was not registered. | Reported | The review was registered in the International Prospective Register of Systematic Reviews (PROSPERO) under registration number CRD420251178394. | Methods, p3 |
| 4b Protocol availability | State where the protocol can be accessed, or state that no protocol was prepared. | Partially reported | The review protocol was registered in PROSPERO; however, a full protocol was not published separately and is not publicly available beyond the registration record. | Methods, p3 |
| 4c Protocol amendments | Describe and explain any amendments to the protocol. | Partially reported | No amendments to the registered protocol were made. This should be explicitly stated in the manuscript if not already included. | Methods, p3 |
| 5 Funding | Describe sources of financial or non-financial support and the role of funders. | Partially reported | The review was funded by the Shanxi Health Commission Project (2025ZYYC011). The specific role of the funder should be clarified, preferably stating that the funder had no role in the design, conduct, analysis, interpretation, or reporting of the review. | Funding statement, p16 |
| 6 Competing interests | Declare any competing interests. | Reported | The authors declare no commercial or financial conflicts of interest. | Conflict of interest statement, p16 |
| 7 Availability of data, code, and materials | Report availability of data, extraction forms, analysis code, and other materials. | Partially reported | The manuscript states that original contributions are included in the article/supplementary material . | Data availability statement, p25 |
| 5. **Introduction** | | | | |
| 8 Rationale | Describe the rationale for the review in the context of existing knowledge. | Reported | The introduction explains the burden of diabetes, the importance of self-management, and the need to evaluate assessment instruments using COSMIN methodology. | Introduction, p2–3 |
| 9 Objectives | State the objective, including outcome domain, population, instruments, and measurement properties. | Reported | The review aimed to evaluate the methodological quality and measurement properties of behavior-related diabetes self-management assessment instruments and to provide evidence-based guidance for instrument selection. | End of Introduction, p3 |
| 6. **Methods** | | | | |
| 10 Guidelines followed | Specify the methods and/or guidelines followed. | Partially reported | The review states that it was reported according to PRISMA and conducted using COSMIN methodology. It would be preferable to explicitly state compliance with the PRISMA-COSMIN for OMIs 2024 reporting guideline. | Methods, p3 |
| 11 Eligibility criteria | Specify inclusion and exclusion criteria. | Reported | The inclusion and exclusion criteria are defined in terms of population, construct, instrument type, measurement property evaluation, language, and publication type. | Methods, p3 |
| 12 Information sources | Specify all databases and other sources searched, including last search date. | Reported | Eight databases were searched from inception to December 15, 2025, and reference lists of included studies were manually screened. | Methods, p3 |
| 13 Search strategy | Provide complete search strategies for all databases or indicate where they are available. | Partially reported | The manuscript provides the search framework and keywords and presents the PubMed strategy in Figure 1. If required by the journal, complete strategies for all databases may be added in supplementary material. | Methods / Figure 1, p3, p25 |
| 14 Selection process | Describe how studies were selected, including number of reviewers and independence. | Reported | Two reviewers independently screened titles, abstracts, and full texts, and disagreements were resolved through discussion or a third reviewer. | Methods, p4 |
| 15 Data collection process | Describe how data were collected, including reviewer number and independence. | Reported | Two reviewers independently extracted data using a standardized form, and disagreements were resolved by discussion or third-party consultation. | Methods, p4 |
| 16 Data items | List and define all extracted data items. | Partially reported | Extracted data included instrument name, first author, publication year, country/region, sample size, number of domains/items, target population, and other key characteristics. This may be strengthened by explicitly listing response format, retest interval, reported measurement properties, evidence quality, and recommendation level. | Methods, p4  Table 1,p20 |
| 17 Risk of bias assessment | Describe methods used to assess risk of bias. | Reported | Risk of bias was appraised independently by two researchers with the COSMIN Risk of Bias Checklist. Consensus was achieved through discussion for inconsistent ratings, and unresolved divergences were adjudicated by a third senior investigator. | Methods, p4 |
| 18 Measurement property assessment | Describe methods used to evaluate measurement property results. | Reported | Measurement properties were evaluated according to COSMIN criteria and rated as sufficient, insufficient, indeterminate, or inconsistent. | Methods, p4 |
| 19a Eligibility for each synthesis | Describe the process used to decide which studies were eligible for each synthesis. | Reported | Evidence was synthesized by instrument and by measurement property; when multiple studies evaluated the same instrument, findings were integrated by property. | Methods, p4 |
| 19b Synthesis methods | Describe methods used to synthesize results. | Reported | Narrative synthesis and COSMIN evidence grading were used; meta-analysis was not performed because of substantial heterogeneity. | Methods / Limitations, pp. 4, 12 |
| 19c Investigation of inconsistency | Describe methods used to investigate causes of inconsistency, if applicable. | Reported | Inconsistency was addressed during evidence grading and interpreted in relation to population differences, retest intervals, statistical methods, and methodological quality. | Methods, p4 |
| 19d Sensitivity analyses | Describe sensitivity analyses, if applicable. | Reported | No sensitivity analyses were conducted because of the limited number of studies per instrument and the substantial heterogeneity of instruments, populations, and reported properties. | Limitations, p15 |
| 20 Certainty assessment | Describe methods used to assess certainty of evidence. | Reported | A modified COSMIN grading approach was used, considering risk of bias, inconsistency, imprecision, and indirectness. | Methods, p4 |
| 21 Recommendations | Describe methods used to make recommendations on instrument suitability. | Reported | COSMIN recommendation levels (Grade A, B, and C) were used to formulate instrument recommendations. | Methods, p4 |
| 7. **Results** | | | | |
| 22a Study selection | Report the number of records identified, screened, excluded, and included, preferably using a flow diagram. | Reported | A total of 3,217 records were identified, 696 duplicates were removed, and 14 studies were included. The screening process is presented in Figure 2. | Results / Figure 2, p5, p25 |
| 22b Excluded studies | Cite excluded studies and explain reasons for exclusion. | Partially reported | The manuscript reports the number of excluded full-text articles and some main exclusion reasons. The study objectives did not align with the current research (n=43); non-Chinese/English literature was included (n=3); mismatched types of assessment tools were used (n=15); and measurement attribute reports were incomplete (n=6). | Results/Figure 2, p5  P25 |
| ,p2523a Characteristics of instruments | Present characteristics of each included outcome measurement instrument. | Reported | Table 1 presents instrument characteristics, including instrument name, country, retest interval, target population, sample size, number of domains/items, and response format. | Results / Table 1, p5, p20 |
| 23b Interpretability | Present interpretability information for each instrument, if applicable. | Partially reported | Interpretability information, such as minimal important change and floor/ceiling effects, was rarely reported in the included studies and therefore could not be systematically synthesized. | Discussion / Limitations, p14–15 |
| 23c Feasibility | Present feasibility information for each instrument, if applicable. | Partially reported | Feasibility was discussed indirectly based on item number, response burden, scoring complexity, and reported completion time where available; however, many feasibility-related aspects were insufficiently reported in the original studies. | Discussion / Table 1,p14, p20 |
| 24 Study characteristics | Present characteristics of each study evaluating one or more measurement properties. | Reported | Table 1 summarizes the included studies in terms of year, country, target population, sample size, retest interval, and instrument characteristics. | Results / Table 1, p20 |
| 25 Risk of bias results | Present risk of bias or methodological quality results for each included study. | Reported | COSMIN methodological quality ratings for each study and measurement property are presented in Table 2 and/or supplementary tables if detailed study-level ratings are moved there. | Results / Table 2, p20-22 |
| 26 Results of individual studies | Present reported results and ratings for each measurement property in each study. | Reported | The Results section reports study-level findings for content validity, structural validity, criterion validity, hypothesis testing, measurement error, responsiveness, internal consistency, and test–retest reliability. | Results, p5-8 |
| 27a Synthesis results | Present all synthesized results and overall ratings. | Reported | Instrument-level measurement property results, evidence quality, and recommendation grades are synthesized in Tables 3 and 4. | Results / Tables 3–4, p22-23 |
| 27b Results of inconsistency investigation | Present findings from the investigation of inconsistency, if applicable. | Reported | Possible sources of inconsistency, such as differences in population, design, retest interval, and analytical methods, are described in the Results and Discussion. | Results / Discussion, p9–11 |
| 27c Sensitivity analysis results | Present results of sensitivity analyses, if applicable. | Not applicable | No sensitivity analyses were performed. | Not applicable |
| 28 Certainty of evidence | Present certainty or confidence in the body of evidence for each measurement property. | Reported | Evidence quality for each measurement property was graded as high, moderate, low, or very low using the modified COSMIN grading approach. | Results, p5–8; Tables 3–4,p22-23 |
| 29 Recommendations | Provide recommendations on suitable instruments for specific uses, if applicable. | Reported | No instrument met Grade A criteria. SDSCA and C-SMOD-A-23 may be cautiously considered for specific populations, but both remain Grade B instruments and require further validation. | Results,p8 |
| 8. **Discussion** | | | | |
| 30a Interpretation of results | Provide a general interpretation of the findings in the context of other evidence. | Reported | The Discussion interprets the overall insufficiency of current evidence, the limited methodological quality of many studies, and the implications of COSMIN recommendation grades. | Discussion, p9–12 |
| 30b Limitations of included evidence | Discuss limitations of the included evidence. | Reported | The Discussion highlights limited content validity evidence, lack of cross-cultural validity, scarce evidence for measurement error and responsiveness, small sample sizes, and incomplete reporting. | Discussion, p9–11 |
| 30c Limitations of the review process | Discuss limitations of the review process itself. | Partially reported | The manuscript notes language restrictions and substantial heterogeneity across studies. This item can be strengthened by clearly stating process-level limitations such as exclusion of non-Chinese/non-English studies and the absence of quantitative meta-analysis. | Discussion / Limitations, p 14-15 |
| 30d Implications for practice, policy, and future research | Discuss the implications of the findings for practice, policy, and future research. | Reported | The manuscript provides implications for instrument selection in clinical and research settings and recommends strengthening content validity, cross-cultural validation, measurement error, responsiveness, and standardized reporting in future studies. | Discussion, p12–15 |
